# Implementation of offering choice of pulmonary rehabilitation location to people with COPD: a protocol for the process evaluation of a cluster randomised controlled trial

**DOI:** 10.1186/s13063-023-07179-2

**Published:** 2023-03-08

**Authors:** Narelle S Cox, Anne E Holland, Arwel W Jones, Christine F McDonald, Paul O’Halloran, Ajay Mahal, Graham Hepworth, Natasha A Lannin

**Affiliations:** 1 Respiratory Research@Alfred, Department of Immunology and Pathology, Central Clinical School, Monash University, Level 6, The Alfred Centre, 99 Commercial Road, Melbourne, VIC 3004 Australia; 2 Institute for Breathing and Sleep, Melbourne, Australia; 3 Physiotherapy, Alfred Health, Melbourne, Australia; 4 Respiratory and Sleep Medicine, Austin Health, Melbourne, Australia; 5 Department of Medicine, University of Melbourne, Melbourne, Australia; 6 School of Psychology and Public Health, La Trobe University, Melbourne, Australia; 7 The Nossal Global Institute for Global Health, University of Melbourne, Melbourne, Australia; 8 Statistical Consulting Centre, University of Melbourne, Melbourne, Australia; 9 Department of Neuroscience, Central Clinical School, Monash University, Melbourne, Australia; 10 Occupational Therapy, Alfred Health, Melbourne, Australia

**Keywords:** Process evaluation, Pulmonary rehabilitation, Implementation, Telerehabilitation, Chronic obstructive pulmonary disease

## Abstract

**Background:**

Pulmonary rehabilitation (PR) is a core component of management people with chronic obstructive pulmonary disease (COPD); yet, people with COPD face significant barriers to attending centre-based PR programs. The emergence of new models of PR, remotely delivered directly into people’s homes, has the potential to improve rehabilitation access and completion by providing patients with a choice of rehabilitation location (centre or home). However, offering patients a choice of rehabilitation model is not usual practice. We are undertaking a 14-site cluster randomised controlled trial to determine whether offering choice of PR location improves rehabilitation completion rates resulting in reduced all-cause unplanned hospitalisation over 12 months. The aim of this paper is to describe the protocol for the process evaluation of the HomeBase2 trial.

**Methods:**

A mixed methods process evaluation, to be undertaken in real time, has been developed in accordance with UK Medical Research Council (MRC) recommendations on process evaluation of complex interventions. This protocol describes the intended use of two theoretical frameworks (RE-AIM framework (Reach; Effectiveness; Adoption; Implementation; Maintenance) and Theoretical Domains Framework (TDF)) to synthesise findings and interpret data from a combination of qualitative (semi-structured interviews) and quantitative (questionnaires, clinical outcome data, intervention fidelity) methodologies. Data will be collected at an intervention, patient and clinician level. Qualitative and quantitative data will be used to derive context-specific potential and actual barriers and facilitators to offering patients choice of rehabilitation location. Acceptability and sustainability of the intervention will be evaluated for future scale-up.

**Discussion:**

The process evaluation described here will appraise the clinical implementation of offering a choice of rehabilitation program location for people with COPD. It will identify and evaluate key factors for future scale-up and sustainability and scale-up of offering choice of pulmonary rehabilitation program model for people.

**Trial registration:**

ClinicalTrials.gov NCT04217330 Registration date: January 3 2020.

## Background

Chronic obstructive pulmonary disease (COPD) is a leading cause of disability and mortality globally [1]. In excess of 350 million people worldwide are estimated to live with COPD [2], which is characterised by distressing breathlessness, physical limitation, poor quality of life and repeated exacerbations requiring hospitalisation [3]. Pulmonary rehabilitation is a comprehensive program of exercise and education for people with COPD that effectively reduces symptoms and improves function and quality of life [4]. Referral to pulmonary rehabilitation is recommended in clinical guidelines [1], and people who complete a program of pulmonary rehabilitation more than halve their likelihood of hospitalisation in the following year [4, 5]. Yet, despite the proven benefits of pulmonary rehabilitation, fewer than 10% of people with COPD ever attend a program [6].

Key barriers to undertaking pulmonary rehabilitation include a lack of qualified staff [7], insufficient programs to serve patient needs [8], low referral rates and poor uptake of program offer by those who are referred [9]. Issues relating to travel and transport to attend a program, carer or work responsibilities and limited understanding of the benefits of pulmonary rehabilitation further impact access to programs [10, 11]. Finding ways to overcome these barriers and to support more people to complete a program of pulmonary rehabilitation are key foci of international respiratory societies [12]. We have previously established that a home-based model of pulmonary rehabilitation (HomeBase), delivered over the telephone, is associated with greater program completion (91% home-based versus 49% centre-based) [5] and achieves equivalent clinical outcomes at similar program delivery costs to centre-based pulmonary rehabilitation [5]. This suggests that offering patients their choice of home or centre-based pulmonary rehabilitation may increase program completion.

While undertaking rehabilitation at their preferred location may help improve rehabilitation completion rates, offering patients a choice of rehabilitation location is not routine clinical practice and has not previously been studied in a clinical trial. Additionally, implementation of home-based models of program delivery into clinical practice is challenging. Prior to the COVID-19 pandemic, fewer than 5% of pulmonary rehabilitation programs offered a home-based service [13], and the majority of services report reverting to centre-based program delivery with easing of COVID-19-related restrictions [14]. This highlights the need to prospectively and systematically understand the processes and behaviours that influence implementation of offering choice of pulmonary rehabilitation location to support future scale-up.

### The HomeBase2 trial: implementing a choice of pulmonary rehabilitation models in COPD

The HomeBase2 trial is an Australian Medical Research Future Fund supported trial. It is a cluster randomised controlled trial at 14 centre-based pulmonary rehabilitation programs in Australia, with programs (clusters) randomised 1:1 to the intervention or control group. A detailed protocol for the HomeBase2 study has been published [15], with trial design elements described briefly here. Intervention clusters offer people with COPD referred to pulmonary rehabilitation their choice of rehabilitation location, either home-based or centre-based. Control clusters will offer only centre-based pulmonary rehabilitation, in accordance with usual practice. Centre-based pulmonary rehabilitation will consist of two sessions per week of supervised, group-based exercise training and may also include self-management training. The home-based program will adhere to our previously established protocol, which has been described extensively [5, 15, 16] and conforms with the recently published essential components of pulmonary rehabilitation [17]. The home-based program comprises a home-visit by a physiotherapist to establish exercise training and ensure safety, followed by seven once-weekly telephone calls with a physiotherapist trained in motivational interviewing to progress exercise training and build capacity for behaviour change [5]. The primary outcome of the HomeBase2 trial is all-cause unplanned hospitalisation over 12 months. Clinical outcomes and healthcare costs will be compared between programs implementing a choice of rehabilitation location (home or centre) and those that offer centre-based only. A total of 490 individuals with a confirmed diagnosis of COPD who consent to participate will be recruited. The recruitment target is 35 participants at each site. Alfred Hospital Ethics Committee has approved the protocols for the main trial and this process evaluation (Project ID 55803, local reference 379–19).

### The process evaluation

As offering a choice of rehabilitation location is a departure from usual practice, and home-based rehabilitation is not widely implemented in clinical practice [18], a process evaluation will be conducted. Process evaluations conducted in tandem with clinical trials provide explanations for how and why interventions are or are not effective, for which participants and in which contexts and circumstances [19]. Trials of complex interventions at multiple sites, such as HomeBase2, where there may be differences in the implementation of the intervention in response to unique local requirements, are well suited to evaluation of process [20]. By assessing intervention delivery, including the perceptions of health professionals regarding the intervention and specific local barriers and facilitators to its implementation, the process evaluation will inform future adoption and/or adaptation of the intervention into wider clinical practice [19]. This paper provides the detailed protocol for the process evaluation embedded in the HomeBase2 trial. The process evaluation and trial outcomes assessment will be conducted in parallel by separate research staff.

### Aims

The aim of the process evaluation conducted alongside the HomeBase2 trial is to support the interpretation of trial outcomes and refine processes for future implementation and scale-up into practice. Therefore, the planned process evaluation is intended to answer the following questions:

1. To what extent did the clinicians or patients use the various components of the planned intervention programs (home-based or centre-based pulmonary rehabilitation)?

2. Which factors are associated with effective implementation of offering choice of pulmonary rehabilitation location?

3. What barriers to and facilitators exist for implementation of the intervention, from the perspectives of the clinicians delivering the program, and the patients who are offered a choice in rehabilitation location?

4. Is offering choice of pulmonary rehabilitation location acceptable to clinicians and patients?

5. How likely is the HomeBase2 intervention to be sustainable and what factors might ensure sustainability?

## Methods

A mixed method process evaluation embedded within the HomeBase2 trial, informed by recommendations of the UK Medical Research Council (MRC) on process evaluation of complex interventions [21], will use two theoretical frameworks (RE-AIM framework (Reach; Effectiveness; Adoption; Implementation; Maintenance) [22] and Theoretical Domains Framework (TDF) [23]) to synthesise findings and interpret data from a combination of qualitative and quantitative methodologies.

**Fig. 1:**
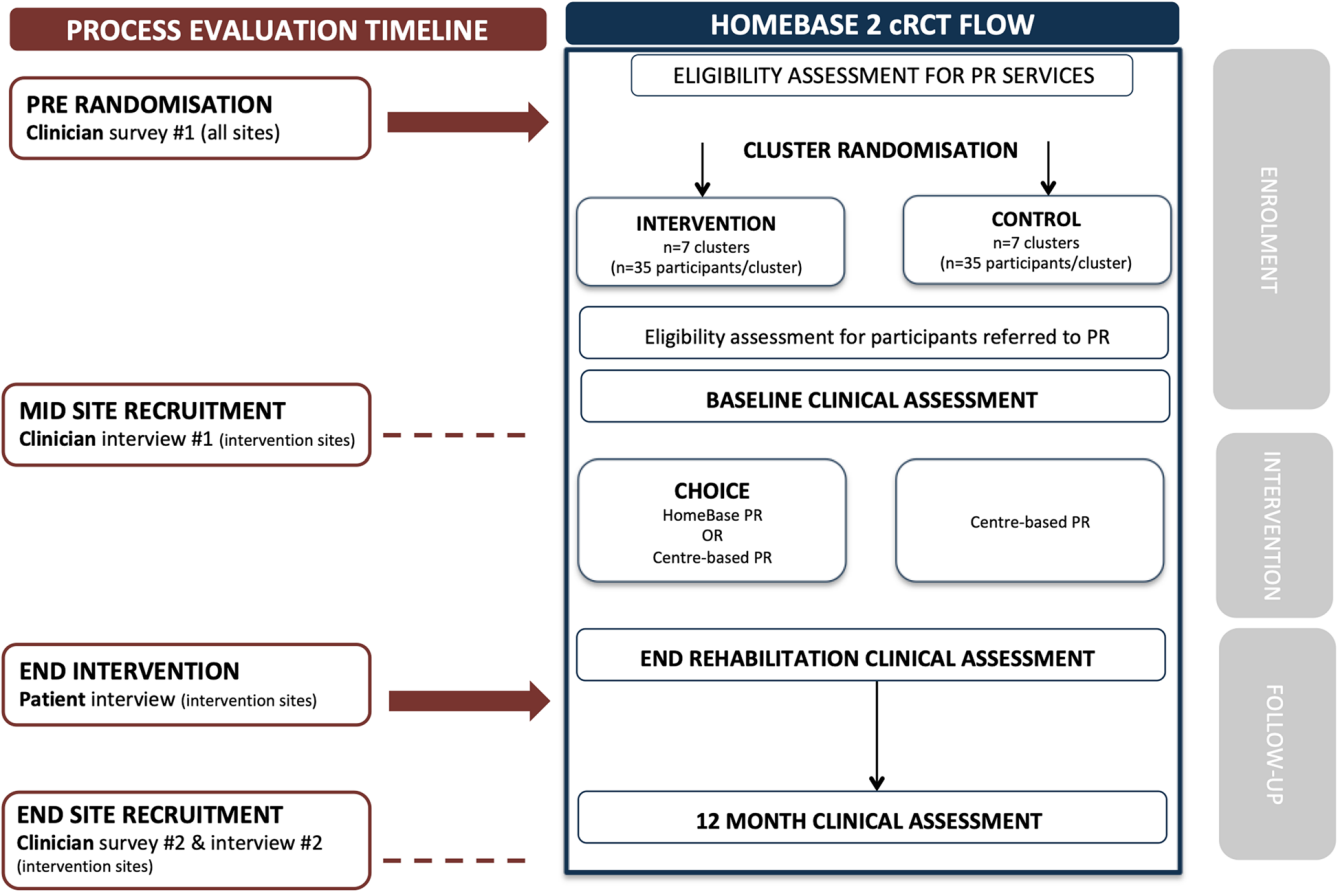
HomeBase2 process evaluation, data collection and timeline

Translation and behaviour change success in intervention clusters will be evaluated using the RE-AIM framework which aims to improve the sustainable adoption and implementation of effective, generalisable, evidence-based interventions [22]. Outcome measures relative to RE-AIM components include intervention participation rates, program completion rates and barriers and facilitators to implementation (Fig. 1). To more comprehensively understand participant perceptions of barriers and facilitators to implementation of the intervention (i.e. aim 3), the TDF [23] will be used as a coding framework. The TDF is an integrative framework that employs behaviour change theories to explain issues relating to implementation of evidence-based practice into clinical care [23, 24].

### Process evaluation design and conduct

A mixed methods study design, with formative evaluation, will be undertaken in real time.

#### Intervention level

Process data will be described quantitatively to determine the fidelity of the intervention (aim 1). All intervention sites will be provided with resources and training by the research team, including a script for describing the rehabilitation programs on offer to inform participant choice, resources and equipment to conduct an initial home-visit for exercise training prescription, specialised training in motivational interviewing to enable delivery of the telephone modules of the home-based rehabilitation program and ongoing support and training from an expert motivational interviewing practitioner. The degree to which the interventions are delivered in accordance with the study protocols will be evaluated and described according to the RE-AIM framework. Outcomes will include the following: proportion of eligible PR referrals offered program choice and intervention participation rates (proportion who choose home-based and centre-based rehabilitation) (*Reach*); program completion (≥ 70% sessions attended) and clinical outcomes (*Effectiveness*); number of staff trained to deliver home-based pulmonary rehabilitation and number of home-visits and MI calls completed (*Adoption*); program components delivered (e.g. exercise training/progression) (*Implementation*); and intent to continue delivery of a home-based program and modifications required for local context (*Maintenance*). Clinical outcomes and site characteristics will also be compared between sites that achieved an average participant rehabilitation completion rate ≥ 70% versus those that did not.

#### Participant level

Evaluation of clinical outcomes for all participants will occur at baseline, end rehabilitation and 12 months after rehabilitation completion and be completed by an assessor blind to group allocation. Patient participants from intervention (‘choice’) clusters will be invited to participate in a guided discussion after the conclusion of their 8-week rehabilitation period and completion of their end-rehabilitation assessment. Discussion will be undertaken to gain an in-depth understanding of patient’s perceptions and experience of the rehabilitation program and the opportunity to choose their rehabilitation location. Qualitative interviews will be led by an experienced researcher who is not involved in providing either pulmonary rehabilitation model for the intervention (‘choice’) clusters nor in providing usual care for participants. Interviews will be conducted via telephone, at a time of the participants’ choosing. See online supplementary material for interview guideline addressing study aims 2–4. A sampling framework will be employed to ensure that data from patient participants reflect all sites (metropolitan and rural; hospital-based programs and community-based programs) as well as key features of individuals commonly referred to centre-based pulmonary rehabilitation (see Table 1). All patient participants will provide written informed consent on entry to the study, which encompasses invitation to undertake a qualitative interview.

**Table 1:** Key participant features to be represented in patient participant interviews

	**Feature**
Trial factors	• State • Hospital outpatient PR program • Community-based PR program
Patient factors	• Chose home-based PR • Chose centre-based PR • Sex • Age (< 75 years versus ≥ 75 years) • Oxygen user • Previously undertaken PR • Lives alone • Currently working • Completed high school education
Disease factors	• FEV_1_ (< 50%predicted versus ≥ 50% predicted) • ≥ 1 hospital admission for COPD in last 12 months • Exercise capacity (baseline 6MWD < 357 m)

*PR* Pulmonary rehabilitation, *FEV*_*1*_ Forced expiratory volume in 1 s, *COPD* Chronic obstructive pulmonary disease, *6MWD* 6-Minute walk distance; m, metres

Patient participant qualitative interviews will be voice-recorded and transcribed verbatim. All participant discussions and responses will be coded to ensure participants cannot be identified from their responses. Two researchers will independently undertake line-by-line thematic analysis of the de-identified transcribed interviews and descriptive codes generated to represent the data [25]. Using a process of repeated reading, and referral back to the de-identified transcripts, two independent sets of descriptive codes will be produced. This reviewing and checking process will ensure the context and intent of participant responses is accurately represented [26]. Descriptive codes will be refined into themes and sub-themes through discussion and illustrated using representative quotes from participants.

#### Clinician level

To explore and explain relationships between intervention implementation, trial outcomes and barriers and facilitators to implementation quantitative and qualitative data will be evaluated (aims 2–5). Clinician participants will be invited to complete an online survey (Qualtrics®, Utah USA) prior to recruitment of each site’s first participant to understand clinicians’ perspectives of the barriers and facilitators to offering choice of pulmonary rehabilitation location. Clinicians at sites randomised to intervention clusters will be invited to complete a follow-up survey at the conclusion of participant recruitment at their site. Survey responses will be coded so that baseline responses can be paired with later survey responses. The questionnaire comprises 41 questions, based on the TDF [24], to be answered on a 6-point Likert scale (completely agree through completely disagree) (Table 2). Additionally, the questionnaire seeks clinicians’ perspectives on delivering alternative models of pulmonary rehabilitation (4 questions) and their comfort with offering choice of rehabilitation location (3 questions). Respondents are offered the opportunity to list specific barriers and facilitators to offering patients a choice of pulmonary rehabilitation location using open text fields. Finally, demographic details relating to clinician professional delegation and experience are collected. Survey data will be reported quantitatively with number and proportion of respondents. Survey responses will be described narratively according to the TDF components contributing to question development [23]. Open-ended questions relating to perceived barriers and facilitators to offering choice of pulmonary rehabilitation location will be coded against the TDF to describe underlying behaviours [23].

**Table 2:** Survey evaluation of perspectives on offering choice of rehabilitation location according to TDF domain

TDF domain	No. questions^a^	Representative question
Knowledge	5	I know that choice of location for delivery of pulmonary rehabilitation can change how many patients complete pulmonary rehabilitation
Skills	3	I have received training in how to deliver HomeBase pulmonary rehabilitation
Social/professional role and identity	3	Only a suitably trained physiotherapist can deliver a HomeBase pulmonary rehabilitation program
Beliefs about capabilities	5	I am confident I could provide the necessary information for a patient to make an informed choice about where they would like to undertake their pulmonary rehabilitation program
Optimism	1	If I offered a patient a choice of pulmonary rehabilitation location, I feel optimistic there would be minimal risk to the patient
Beliefs about consequences	6	For me, patients exercising independently with minimal supervision at home places patients at risk
Reinforcement	3	Whenever I look for ways to improve pulmonary rehabilitation access, I get recognition from professionals who are important to me
Intentions	3	I will definitely speak with the management of my organisation about adopting a ‘choice’ model of pulmonary rehabilitation delivery
Goals	1	I have a clear plan for how I will implement choice of pulmonary rehabilitation location in my service
Memory, attention, decision processes	1	I am familiar with the content of the Australian and New Zealand Pulmonary Rehabilitation Guidelines
Environmental context and resources	6	In the organisation that I work, there is enough time to develop a new pulmonary rehabilitation service delivery option
Social influences	4	My colleagues are supportive of offering a choice for pulmonary rehabilitation location
Emotion	2	I feel nervous about offering the patient a choice of pulmonary rehabilitation location
Behavioural regulation	1	I have a clear plan for how I will implement choice of pulmonary rehabilitation location in my service

*TDF* Theoretical domains framework

^a^Total number of survey questions is 41. Any question may be representative for more than 1 TDF domain

Barriers and facilitators to successful implementation will also be explored through qualitative interviews with clinicians from intervention clusters. Clinicians at sites randomised to intervention clusters will be invited to participate in 1:1 qualitative interviews at a time corresponding to approximately 50% of their site recruitment and at the completion of their site recruitment. The interview discussion guide is designed to understand the clinician experience of offering a choice of pulmonary rehabilitation model, and the experience of delivering an alternative model of pulmonary rehabilitation (if applicable). By seeking clinician experiences at two time points, we will be able to describe perceived and actual barriers to and facilitators for offering/implementing more than one model of pulmonary rehabilitation in the context of any adaptations made as the trial progresses. In addition, we will be able to describe the expectations of the clinicians with respect to offering a choice of pulmonary rehabilitation location, whether these expectations were met by the conclusion of the trial, and perceived acceptability of the intervention (aim 4). Insights from the second clinician data collection time point will additionally be drawn together with recruitment data and survey feedback at the end of the trial to determine factors which might ensure sustainability of the HomeBase2 intervention at trial completion (aim 5).

#### Logic model

**Fig. 2:**
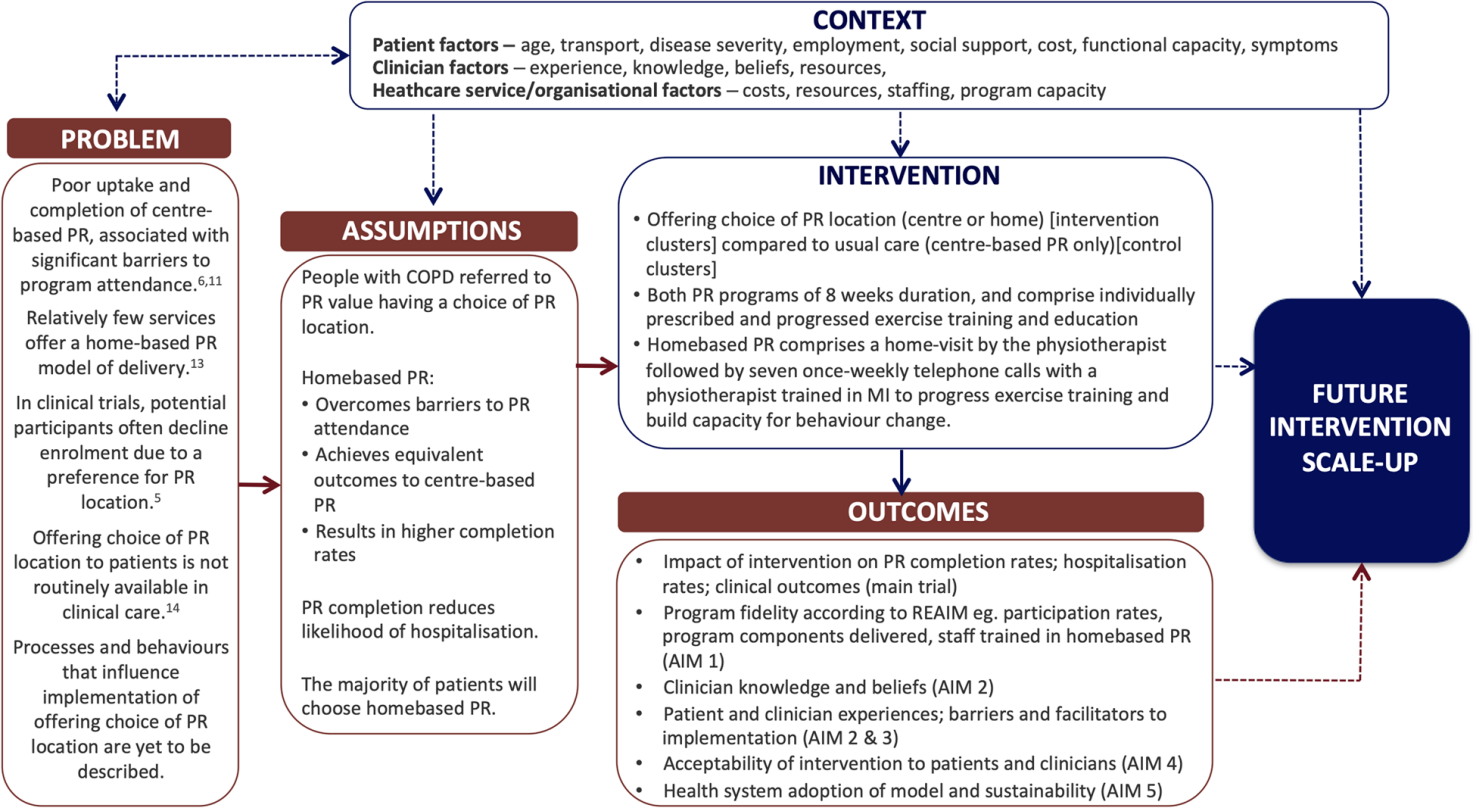
Logic model for the HomeBase2 implementation trial

In keeping with UK MRC guidance [21], a logic model (Fig. 2) was created that was informed by the underlying evidence, the researchers experience of home-based pulmonary rehabilitation and key factors that could potentially influence implementation.

### Ethics and dissemination

The design of this process evaluation is covered within the ethics application and overall protocol of the HomeBase2 trial approved by the Alfred Health Human Research Ethics Committee (local reference 379/19). Local governance approvals were obtained from all participating sites. Written informed consent will be obtained from all participants (patient and clinician). The results of this process evaluation will be submitted for publication in peer-reviewed journals, presented at conferences and described in lay communications.

### Patient and public involvement

Patient and public involvement in the HomeBase2 trial has been from trial inception, including study design and trial oversight. Full details are described elsewhere [15].

## Discussion

In response to restrictions associated with the COVID-19 pandemic, there was a rapid shift from centre-based pulmonary rehabilitation services to those that could be delivered remotely [27]. However, despite what appeared to be an ideal environment for alternative models of pulmonary rehabilitation delivery, most centres *reverted back* to traditional, centre-based, in-person delivery models as soon as this was possible [14]. This suggests that, as providing the option of home-based rehabilitation programs is a significant departure from usual clinical practice, it requires clinician, health system and policy adaptations to ensure sustainable delivery of effective, evidence-based programs. In addition, healthcare professionals have expressed apprehension relating to skills and technology that may be required for the delivery of alternative models of pulmonary rehabilitation [28], and up to 40% of patients are not interested in using alternative means such as the telephone or internet to receive pulmonary rehabilitation [29, 30]. This highlights the challenges of implementing alternative models of pulmonary rehabilitation into clinical practice. 

## Trial status

Recruitment for the HomeBase2 trial commenced in March 2021 and remains ongoing. Trial recruitment is anticipated to be completed by April 2024.

## Data Availability

The coordinating principal investigator (Prof Anne Holland) will have access to the final trial dataset. Any requests for data to support the protocol can be made to the principal investigator (Prof Anne Holland). The anonymised datasets analysed during the current study will be available from the corresponding author on reasonable request, and on attainment of relevant approvals from the overseeing ethics committee, as is the full protocol.

## Abbreviations

COPD: Chronic obstructive pulmonary disease
PR: Pulmonary rehabilitation
RE-AIM: Reach, Effectiveness, Adoption, Implementation, Maintenance (framework)
TDF: Theoretical Domains Framework
